# Inhaled corticosteroids for COVID-19: a real-world data analysis on guideline adherence

**DOI:** 10.3399/BJGPO.2024.0135

**Published:** 2025-04-24

**Authors:** Jasper WA van Egeraat, Ton Kuijpers, Jako Burgers, Hendrikus van Os, Niels H Chavannes, Tobias N Bonten

**Affiliations:** 1 Department of Public Health and Primary Care, National eHealth Living Lab, Leiden University Medical Center, Leiden, Netherlands; 2 Dutch College of General Practitioners (NHG), Utrecht, Netherlands; 3 Department Family Medicine, Maastricht University, Maastricht, Netherlands

**Keywords:** COVID-19, electronic health records, General practice, guideline adherence, SARS-CoV-2

## Abstract

**Background:**

The recommendation to consider prescribing inhaled corticosteroids (ICSs) to a subgroup of vulnerable patients with COVID-19 was added to the Dutch medical guideline on 2 November 2021, and was also adopted by other countries during the pandemic.

**Aim:**

To evaluate the adherence of GPs to this guideline, and whether the quality of real-world data is sufficient to study the effect of revised guidelines on prescribing behaviour.

**Design & setting:**

A retrospective cohort study using Dutch primary care data from the Extramural Leiden University Medical Center (LUMC) Academic Network database, containing patient data of 129 general practices in the Leiden – The Hague area.

**Method:**

We performed an interrupted time series analysis to measure the effect of the new recommendation on the prescription rate of ICSs, accounting for general trends and seasonal fluctuations.

**Results:**

Between 1 July 2020 and 1 August 2022, 131 482 patients had 164 098 COVID-19 consultations. During this period, 1709 patients received 2094 ICS prescriptions for COVID-19. After the guideline update, there was an instantaneous decrease in prescription rate (incidence risk ratio [IRR] 0.47, 95% confidence intervals [CI] = 0.32 to 0.69). Prescription rate in the subgroup of vulnerable patients did not change significantly (IRR 0.93, 95% CI = 0.66 to 1.32), while less vulnerable patients were prescribed significantly fewer ICSs (IRR 0.29, 95% CI = 0.14 to 0.59).

**Conclusion:**

The revision to the COVID-19 guideline had significant impact on GPs’ prescribing behaviour soon after publication: prescription rate remained constant for vulnerable patients, while less vulnerable patiens received ICS prescriptions significantly less often. Using electronic health records it is feasible to assess changes in guideline adherence using interrupted time series.

## How this fits in

Although guideline adherence is not a new research topic, the effects of revisions to guidelines have not been widely studied. The method of interrupted time series is a natural way to assess changes in guideline adherence when revisions are made. We found that electronic health record quality is sufficient to study the effect of the November 2021 update to the Dutch COVID-19 guideline, which advised GPs to consider prescribing inhaled corticosteroids to vulnerable patients.

## Introduction

During the COVID-19 pandemic, relatively few patients with chronic respiratory diseases, such as asthma or chronic obstructive pulmonary disease (COPD), were hospitalised. This was in contrast to expectations, as these patients were more susceptible to a COVID-19 infection and would have had a higher mortality than patients without comorbidities.^1^ Patients with chronic respiratory disease are frequently prescribed inhaled corticosteroids (ICSs) such as budesonide.^2,3^ It was hypothesised that ICSs protect patients against severe COVID-19, leading to several randomised clinical trials evaluating the effect of ICSs on COVID-19 susceptibility, severity, and mortality. It was found that inhaled budesonide reduces the time to recovery after COVID-19 in patients with chronic lung disease.^4–7^

After considering the aforementioned evidence, healthcare organisations in several countries recommended budesonide as a treatment for COVID‑19. This recommendation was adopted in medical guidelines in India, Russia, Saudi Arabia, and British Colombia in Canada, among others.^8–11^ In the Netherlands, the Dutch College of General Practitioners (NHG) updated its COVID-19 guideline on 2 November 2021, issuing a recommendation for GPs to consider prescribing budesonide to older patients with recent symptoms, insufficient vaccine protection, and/or comorbidities. Before the guideline update there was no recommendation regarding budesonide or other ICSs for patients with COVID-19. The guideline update was highlighted in the NHG newsletter the same week.

After randomised clinical trials provide new evidence, the translation, incorporation, and implementation of this new evidence into new guidelines and recommendations is often a slow process.^12^ Furthermore, it remains in question whether new or updated guidelines are followed, which could be a deliberate choice by clinician or patient, or unintentional due to unfamiliarity with the latest recommendations. Research using real-world data is essential to assess whether guidelines are being followed.^13,14^

This study aims to analyse the change in prescription rate for budesonide and other ICSs after the guideline update for two reasons. Firstly, to observe whether — and to what extent — ICSs have been prescribed to patients with COVID-19 after the guideline revision. Secondly, to assess whether current real-world data is of sufficient quality to study changes in prescribing behaviour.

## Method

### Data source and patient population

We used electronic health records collected by GPs affiliated with the Extramural Leiden University Medical Center Academic Network (ELAN), Leiden, the Netherlands.^15^ The ELAN data warehouse contains anonymised data including patient characteristics, consultations, and medication prescriptions from patients living in the urban cities of Leiden, the Hague, and Zoetermeer and the surrounding suburban and rural areas. In 2021, 572 930 patients out of the total 1 719 617 individuals in this region (33%) were registered with one of the ELAN GPs.^16^ Diagnoses and symptoms of consultations are coded according to the International Classification of Primary Care (ICPC).^17,18^ Medication is coded according to the Anatomical Therapeutic Chemical (ATC) classification.^19^

Our study population consisted of all patients enlisted with ELAN general practitioner centres who had at least one consultation (ICPC R83 or R83.03) for COVID-19 within the period of 1 July 2020 to 1 August 2022. Initially, R83 was used to classify COVID-19 cases before the specific code R83.03 was available.^20,21^ We extracted budesonide prescriptions (ATC R03BA02), as well as other glucocorticoids (ATC R03BA*) and corticosteroids in combination with adrenergics or other drugs (ATC R03AK*).

According to the NHG guideline, from 2 November 2021 onwards, patients with COVID-19 were eligible for budesonide if they met all of the following criteria:

- they had symptoms for less than 14 days;
- had not been fully vaccinated, were vaccinated but considered a non-responder, or vaccinated but developed moderate to severe complaints;
- and were aged 65 years and over, or aged 50 years and over with at least one of the following comorbidities: (severely) reduced immune system, (severe) chronic kidney damage, cardiovascular disease, diabetes mellitus, COPD, cirrhosis of the liver and morbid obesity.^22^

Information on symptom duration, vaccinations, and obesity status were unavailable in the ELAN database. ICPC codes of comorbidities are given in Supplementary Table 1.

Approximately 25% of prescriptions were coupled to a diagnosis. Thus, for the majority of ICS prescriptions we could not determine with certainty whether it was prescribed for COVID-19 or for chronic respiratory diseases. Therefore, we assumed that any ICS prescription within 14 days of a COVID-19 consultation was prescribed for this reason.

To preserve patient anonymity, the ELAN data warehouse only provides birth year, rather than birth date. Patient age was estimated by assuming a birth date of 2 July in the birth year. All other required variables were complete.

### Study design and statistical analysis

We used an interrupted time series design to assess ICS prescription rate for patients with COVID-19 before and after the guideline was published. Our methodology adhered to recommendations outlined by Lopez *et al* in 2016.^23^ For the time series, we extracted the number of COVID-19 consultations with ICS prescription and the total number of COVID-19 consultations in every week of the study period, from which we determined the weekly rate of prescription. The interruption was set as the day the COVID-19 guideline was updated to include the advice on budesonide, on 2 November 2021. The study included 70 weeks of pre-intervention data and 39 weeks of post-intervention data.

To estimate the effect of the interruption on the prescription rate, we fitted a quasi-Poisson model to the weekly counts of COVID-19 episodes, with the weekly total COVID-19 episodes included as offset. We included a dummy variable which was 0 in the weeks before the guideline update, and 1 afterwards. Seasonal fluctuations were accounted for by including dummy variables for the year quarters in the model. The trend over the study period and trend change was modelled with variables indicating the number of weeks since the start of the study and the number of weeks since the update (equal to 0 before the update). A quasi-Poisson model was chosen to adjust for overdispersion by scaling the standard errors.^23^ We tested for auto-correlation using the Durbin Watson test, and compensated for any auto-correlation by scaling the standard errors of the model with the Newey-West method.^24^ The model formula is specified in the Supplementary materials.

The effect of the interruption was estimated by incidence risk ratios (IRRs) with 95% confidence intervals (CIs) for the instantaneous difference and the difference in trend of the prescription rate. The IRR represents the ratio of the predicted prescription rate for one unit increase in the corresponding covariate, holding other variables constant.

Data management and analysis was done in R software (version 4.3.3) with packages data.table, lubridate, ggplot2, table1, car, lmtest, and sandwich.^25–32^

### Sensitivity and subgroup analysis

As a sensitivity analysis, we fitted the model on the overall study population without correcting for seasonal effects. To perform a subgroup analysis, patients were grouped by whether they satisfied criterion (iii) as given by the NHG or not; patients aged 65 years and over, or 50 years and over with a comorbidity were categorised as vulnerable patients, all other patients were categorised as less vulnerable patients.

## Results

Between 1 July 2020 and 1 August 2022, 131 482 patients together had 164 098 COVID-19 episodes. The mean age of a patient during their first COVID-19 episode was 36.8 years (standard deviation [SD] 20.5 years), and 60 623 (46.1%) subjects were male.

The baseline characteristics of patients who had at least one COVID-19 consultation during the study period are presented in Table 1. There were no significant differences between the patients who were prescribed an ICS before and after the guideline revision. Patients who were prescribed ICSs were more often female, older, with comorbidities, and experienced more distinct COVID-19 episodes, compared with patients who were not prescribed these medications (*P*<0.001 for all comparisons).

**Table 1.** Baseline characteristics of patients who had at least one COVID-19 consultation during the study period

	Prescribed			Not prescribed
Characteristic	Before update, *N*=526	After update, *N*=1208	Overall, *N*=1709	Overall, *N*=129 773
Male sex, n (%)	193 (36.7)	441 (36.5)	627 (36.7)	59 996 (46.2)
Mean age, years (SD)	51.5 (17.2)	48.7 (19.9)	49.6 (19.2)	36.8 (20.4)
Target NHG advice (age ≥65 years or age ≥50 years with comorbidity^a^), n (%)	225 (42.8)	475 (39.3)	689 (40.3)	17 637 (13.6)
Number of episodes, mean (SD)	2.07 (0.8)	1.50 (0.7)	1.66 (0.8)	1.24 (0.5)
Any of the following comorbidities, n (%)	320 (60.8)	718 (59.4)	1019 (59.6)	17 556 (13.5)
Asthma	251 (47.7)	575 (47.6)	808 (47.3)	8464 (6.5)
COPD	42 (8.0)	109 (9.0)	151 (8.8)	1412 (1.1)
Cardiovascular disease	71 (13.5)	170 (14.1)	239 (14.0)	6837 (5.3)
Diabetes	4 (0.8)	7 (0.6)	11 (0.6)	402 (0.3)
Chronic kidney damage	31 (5.9)	67 (5.5)	98 (5.7)	2692 (2.1)
Liver cirrhosis	11 (2.1)	13 (1.1)	23 (1.3)	674 (0.5)

Age and whether the patient is targeted by the NHG advice is computed at the moment of their first COVID-19 episode. There were 25 patients who were prescribed ICSs both before and after guideline update. These are included in both column two and three.

a Comorbidities are asthma, COPD, cardiovascular disease, diabetes, chronic kidney damage, or liver cirrhosis.

During the study period, 1709 patients received a total of 2094 ICS prescriptions following a COVID-19 episode. Budesonide was prescribed 260 times (0.2% of COVID-19 episodes), other glucocorticoids were prescribed 762 times (0.5%), and corticosteroids in combination with adrenergics or other drugs were prescribed 1072 times (0.7%).

**Figure 1.**
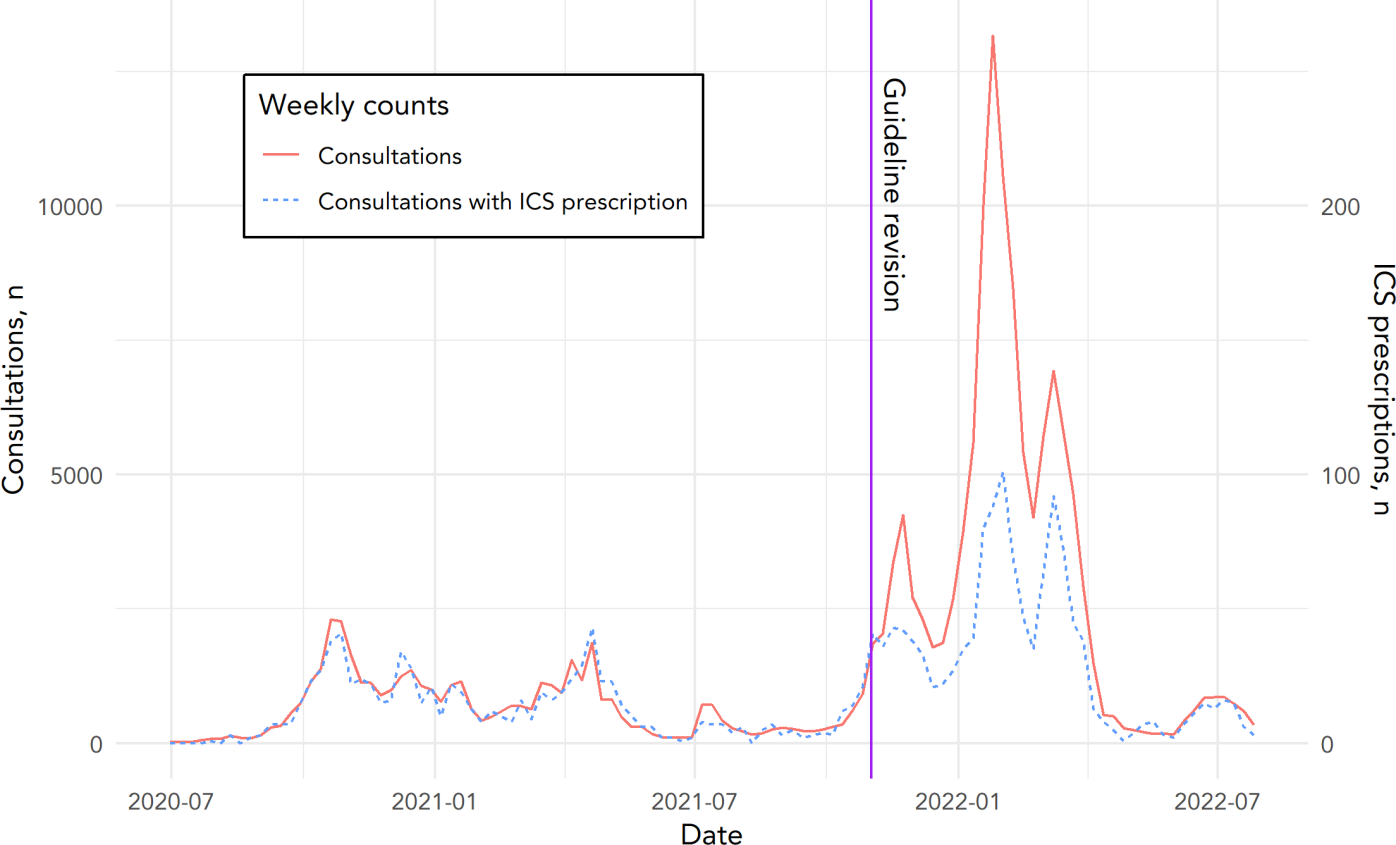
Weekly counts of COVID-19 consultations and COVID-19 consultations with ICS prescriptions. The consultations with ICS prescription follow the scale on the right side of the figure. This scale is chosen such that frequency patterns can be assessed simultaneously. The intervention date was 2 November 2021 (the day the NHG published its updated guideline) with regard to budesonide and is marked with the vertical line

In Figure 1, the number of weekly COVID-19 consultations and weekly consultations with ICS prescription are shown, along with a vertical line indicating the timepoint when the recommendation to consider prescribing ICSs was introduced.

In Table 2, and more extensively in Supplementary Table S2, the results of the fitted quasi-Poisson model are presented. We found a statistically significant decrease in the weekly rate of ICS prescriptions for COVID-19 consultations after publication of the guideline update (IRR 0.47, 95% CI = 0.32 to 0.69). There was no significant trend during the study period (IRR 1.00, 95% CI = 1.00 to 1.01) and no significant trend change after the updated guideline (IRR 1.01, 95% CI = 0.99 to 1.02). Thus, after adjusting for overall trend and seasonal effects, the prescription rate reduced by a factor of 0.47 after guideline revision. The results of the Durbin Watson test, shown in Supplementary Table S3, indicate a minor but significant autocorrelation, for which we adjusted the standard errors and confidence intervals using the Newey-West method.

**Table 2.** Estimated incidence risk ratios for quasi-Poisson model for the weekly prescription rate

Variable	IRR	95% **CI**	p-value
Weeks after study start	1.00	1.00 to 1.01	0.06
After guideline revision (indicator)	0.47	0.32 to 0.69	<0.001
Weeks after guideline revision	1.01	0.99 to 1.02	0.31
Q1 (indicator)	0.70	0.58 to 0.84	<0.001
Q2 (indicator)	1.01	0.81 to 1.27	0.91
Q3 (indicator)	0.75	0.59 to 0.96	0.02

Weeks after study start represents the trend before guideline revision. After guideline revision (indicator) represents the immediate effect of guideline revision and the row for the weeks after guideline revision represents the trend change with respect to the period before revision. Q1, Q2, and Q3 model seasonal effects in the first, second, and third quarter of the year with respect to the fourth quarter.

Autocorrelation has been accounted for by adjusting the confidence interval and p-value using the Newey West method.

CI = confidence interval. IRR = incidence risk ratio.

**Figure 2.**
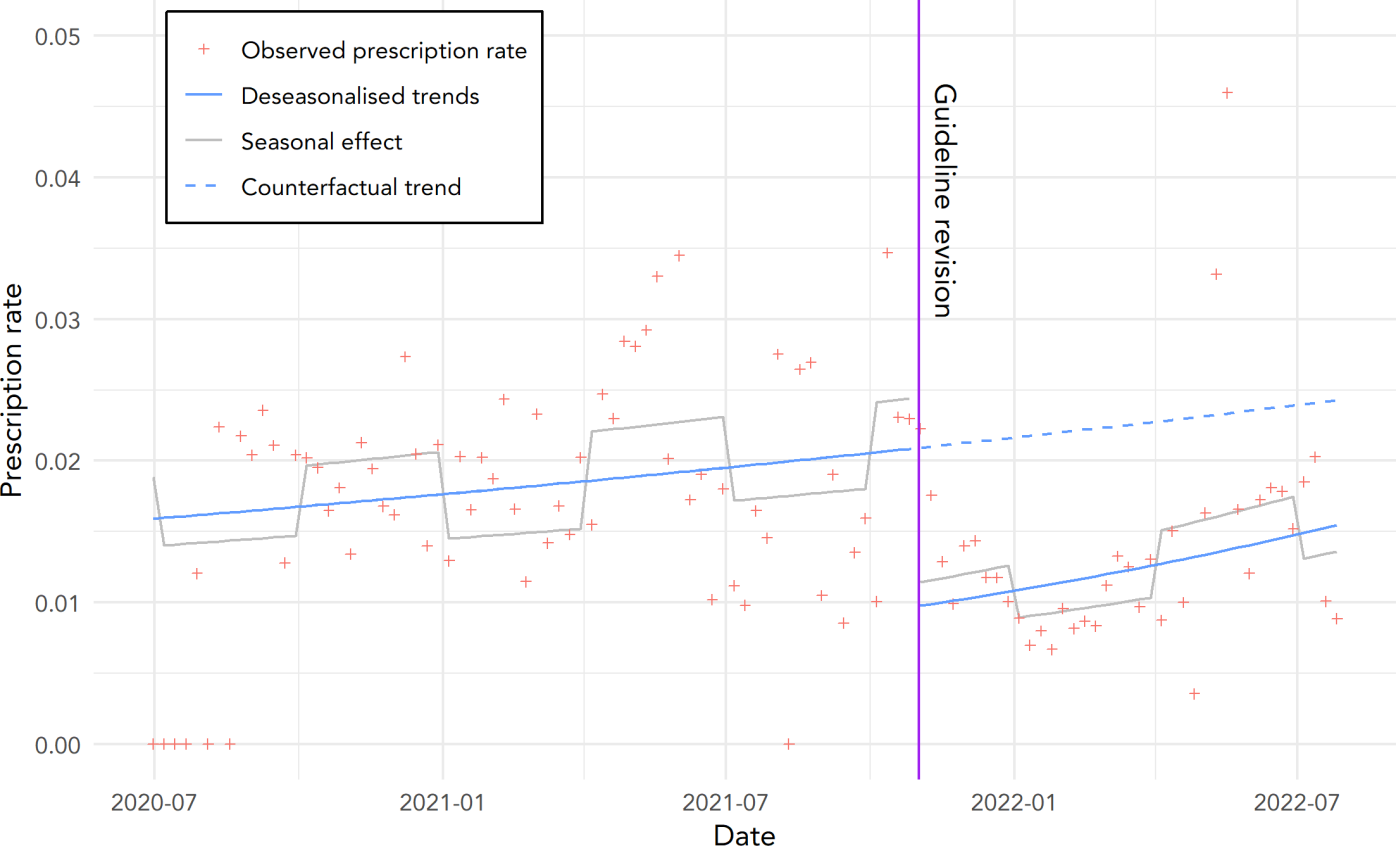
The observed weekly prescription rate (ratio of COVID-19 cases with ICS prescription per COVID-19 consult), along with the trend and seasonal effect. The counterfactual line is formed by continuing the trend estimated from the period before the guideline revision. The vertical line denotes the day of the guideline revision, 11 November 2021

In Figure 2 the weekly rate of COVID-19 cases with ICS prescription per COVID-19 case is presented, along with the model fit and counterfactual trend, which is determined by extending the pre-intervention trend into the post-intervention period.^33^

Our sensitivity analysis shows that the effect without adjusting for the seasonal effects is similar (Supplementary Table S4), but due to higher autocorrelation (Supplementary Table S5) the confidence interval is larger.

We did not detect an instantaneous change in prescription rate for the subgroup of vulnerable patients (IRR 0.93, 95% CI = 0.66 to 1.32), but the prescription rate significantly decreased per week after guideline revision (IRR 0.98, 95% CI = 0.96 to 0.99). In the subgroup of less vulnerable patients we found an instantaneous significant decrease in prescription rate (IRR 0.29, 95% CI = 0.14 to 0.59), but afterwards the rate increased per week (IRR 1.04 95% CI = 1.01 to 1.07). See also Supplementary Tables S6 and S8. In Supplementary Tables S7 and S9 , we report the results of the Durbin Watson tests for the subgroup analyses, which again indicate minor but significant autocorrelation.

## Discussion

### Summary

We found a decrease in the ICS prescription rate for COVID-19 infection after the guideline update, with no significant trend or trend change. The subgroup of vulnerable patients showed a gradual decrease in prescription rate after guideline revision. In the subgroup of less vulnerable patients there was a strong instantaneous decrease in prescription rate, followed by a gradual increase.

Several factors may explain the decrease in overall prescription rate after the guideline change. The NHG advised GPs to consider prescribing ICSs to a specific subgroup of patients, leading to fewer ICS prescriptions in patients outside this group. Our subgroup analysis confirms this result. Another possible reason for the overall decrease in prescription rate, is because of the strength of the recommendation; GPs were advised to *consider* prescribing ICSs to a specific subgroup, rather than being advised to always prescribe.

The extraordinary circumstances of the early phase of the pandemic may have caused GPs to prescribe medication more frequently despite limited evidence. As COVID-19 knowledge increased, GPs prescribing behaviours may have changed to a more cautious approach, decreasing the prescription rate compared with the early phase.

The wave of COVID-19 cases that took place immediately after the guideline update (November 2021) may have also decreased the prescription rate. Due to increased COVID-19 consultations, GPs became more overloaded with work and had less time per patient.^34,35^ To properly instruct patients on how to use inhalers, GPs would need to see them face to face. There may have been a higher proportion of remote consultations during this wave, which would lower the opportunities to prescribe ICSs. Additionally, patients were having relatively milder symptoms in the later stages of the COVID-19 pandemic, due to higher vaccination rates and less severe COVID-19 variants.^36,37^

It seems paradoxical that prescribing behaviour shifted opposite to the recommendation: instead of an increase, the guideline led to an overall decrease in prescriptions. However, subgroup analysis shows the reduction mostly occurred in the group for which the advice is *not* to consider prescribing. Thus, we can conclude that GPs followed the recomendation accurately.

### Comparison with existing literature

In this study we observed the GP prescribing behaviour after a guideline change, supported by evidence from two randomised clinical trials. In the PRINCIPLE trial, 1073 COVID-19 patients aged 65 years and over, or 50 years and over with comorbidities were randomisd to budesonide between 27 November 2020 and 31 March 2021.^7^ In our study population, only 42% of the ICS-prescribed patients fell into this category; they tended to be younger and often without comorbidities. The STOIC trial, with 73 participants assigned to budesonide between 16 July and 9 December 2020, had no specific restrictions on patient characteristics.^6^ The patients of STOIC are similar to the population in our study. Since our study period lasted from 1 July 2020 to 1 August 2022, it is likely that during this time, different COVID-19 variants were dominant compared with the dominant variants of the PRINCIPLE and STOIC trials. Milder variants may have reduced the need for prescribing ICSs.

Previous studies have used real-world data to monitor guideline adherence. For example, natural language processing algorithms have been employed on clinical notes of patients with asthma to assess adherence to asthma guidelines^38^, and a web-based dashboard monitored adherence to an endometrial cancer guideline using a national registry.^39^ As another example, COPD guideline adherence, especially regarding ICS therapy, was studied using data from 900 general practitioners in Italy.^40^ Splitting the study period into four different cohorts allowed the assessment of changes in adherence. Thus, monitoring guideline adherence is feasible with real-world data and various studies have already done so.

### Strengths and limitations

This is the first study that assessed the adherence to the revised guideline of COVID-19 with respect to prescribing ICSs. The strength of our study lies in the use of real-world data of a large number of COVID-19 patients. Our methods and results can be used for future updates to guidelines, not only for COVID-19 but for a wide range of diseases and their guidelines.

A segmented Poisson regression for an interrupted time series design is a scientifically valid method to quantify the effect of changes in guidelines. Its advantage over other methods lies in its relatively simple application with interpretable results, and possibility to incorporate trends and seasonal fluctuations.

A limitation of this study is that the guideline revision was not the only ‘interruption’ within the time period. The study population evolved throughout the study period due to vaccination strategies prioritising older and high risk individuals. Additionally, the dominant COVID-19 variant changed throughout the study period, which could have affected ICS prescription behaviour. Another possible limitation is that the update of the guideline may not be seen as an isolated intervention at one time point. News in the media about the role of ICSs in the treatment of COVID-19 may have influenced clinicians before the updated guideline was officially released, and on the other hand, clinicians may only learn about the guideline revision weeks after the update. Due to these additional interruptions, we may have overestimated the instantaneous effect of the update.

Our data quality, while sufficient in sample size, lacked details such as vaccination status and symptom duration, which would be required to strictly measure the adherence to the COVID-19 guideline. Since information on these factors was unavailable, we might have overestimated the percentage of patients that satisfied the NHG criteria. Additionally, the advice to prescribe an ICS was only to be considered, rather than a ‘must follow’ guideline, complicating assessment of guideline adherence. We only have data on prescriptions, but no information on whether clinicians *considered* prescribing, like the guideline recommended. The relatively weak wording in the guideline and numerous conditions a patient must satisfy, may explain the significant decrease in overall prescription rate after the guideline changed hereto.

Whether medication had been prescribed specifically for COVID-19 or for another disease was unknown for most patients, possibly resulting in an overestimation of prescriptions for COVID-19. By selecting prescriptions occurring within 14 days of a COVID-19 consultation we largely minimised this issue.

### Implications for research

As of 28 November 2023, the recommendation to consider prescribing an ICS has been withdrawn from the Dutch COVID-19 guideline. The reason is that the benefit of ICSs in adults with the omicron variant of COVID-19 and with previous immunity may be smaller and no longer clinically relevant compared with earlier variants of COVID-19.^22^ A future study could assess whether this recommendation change has further decreased the prescription rate.

Our study shows that routine care data can be efficiently used to study behaviour change after guideline changes, which is a time-efficient approach to evaluate an aspect of guideline implementation. Once a guideline has been revised, it is an open question as to how to best communicate these changes to clinicians. In future studies, these methods can be used to optimise and to measure the effect of different dissemination strategies of guidelines.

## Data

The data that support the findings of this study are available from ELAN but restrictions apply to the availability of these data, which were used under license for the current study, and thus are not publicly available. Data are however available from the authors upon reasonable request and with permission of ELAN.
